# Supplementary material for: Decoys provide a scalable platform for the identification of plant E3 ubiquitin ligases that regulate circadian function
Source: eLife. 2019 Apr 5;8:e44558. doi: 10.7554/eLife.44558 (PMC6483598; doi:10.7554/eLife.44558)
Supplement: Transparent reporting form [file elife-44558-transrepform.docx]

***eLife’s* transparent reporting form**

We encourage authors to provide detailed information *within their submission* to facilitate the interpretation and replication of experiments. Authors can upload supporting documentation to indicate the use of appropriate reporting guidelines for health-related research (see [EQUATOR Network](http://www.equator-network.org/%20)), life science research (see the [BioSharing Information Resource](https://biosharing.org/)), or the [ARRIVE guidelines](http://www.plosbiology.org/article/info:doi/10.1371/journal.pbio.1000412) for reporting work involving animal research. Where applicable, authors should refer to any relevant reporting standards documents in this form.

If you have any questions, please consult our Journal Policies and/or contact us: [editorial@elifesciences.org](mailto:editorial@elifesciences.org).

**Sample-size estimation**

- You should state whether an appropriate sample size was computed when the study was being designed
- You should state the statistical method of sample size computation and any required assumptions
- If no explicit power analysis was used, you should describe how you decided what sample (replicate) size (number) to use

Please outline where this information can be found within the submission (e.g., sections or figure legends), or explain why this information doesn’t apply to your submission:

Based on our previous studies we showed that N of 20 was sufficient to detect major and most minor changes in circadian period for our luciferase studies. Due to the magnitude the screening we were undertaking, in combination with the plate size and number of plates that we could do simultaneously, made this number feasible.

This information can be found in the materials and methods section under the sub-header “phenotypic screening.”

**Replicates**

- You should report how often each experiment was performed
- You should include a definition of biological versus technical replication
- The data obtained should be provided and sufficient information should be provided to indicate the number of independent biological and/or technical replicates
- If you encountered any outliers, you should describe how these were handled
- Criteria for exclusion/inclusion of data should be clearly stated
- High-throughput sequence data should be uploaded before submission, with a private link for reviewers provided (these are available from both GEO and ArrayExpress)

Please outline where this information can be found within the submission (e.g., sections or figure legends), or explain why this information doesn’t apply to your submission:

Due to performing the luciferase imaging experiments in the T1 generation, each seedling serves as a biological repeat, and we performed technical repeats (at least two sets of 20, done on different dates or with different vectors) for the majority of genotypes, including all those which we name as “major” regulators. Experiments were excluded from analyses if the variation in the control was greater than 0.75 hours, to control for environmental fluctuations.

For the qPCR experiments, samples were collected in triplicate and processed separately.

This information can be found in the materials and methods section under the sub-headers “phenotypic screening,” and “Measurement of Circadian Gene Expression in *pub59/pub60* mutant lines,” as well as in the results section under “Screen Design.”

**Statistical reporting**

- Statistical analysis methods should be described and justified
- Raw data should be presented in figures whenever informative to do so (typically when N per group is less than 10)
- For each experiment, you should identify the statistical tests used, exact values of N, definitions of center, methods of multiple test correction, and dispersion and precision measures (e.g., mean, median, SD, SEM, confidence intervals; and, for the major substantive results, a measure of effect size (e.g., Pearson's r, Cohen's d)
- Report exact p-values wherever possible alongside the summary statistics and 95% confidence intervals. These should be reported for all key questions and not only when the p-value is less than 0.05.

Please outline where this information can be found within the submission (e.g., sections or figure legends), or explain why this information doesn’t apply to your submission:

The methods section under the subheaders “Data Normalization and Statistical Analysis,” “Measurement of Circadian Gene Expression in pub59/pub60 mutant lines”, and “Measurement of PRR9 spliceoforms” contains explanations of the statistical tests used and analysis performed.

The exact p-values and summary statistics can be found in Table S3.

(For large datasets, or papers with a very large number of statistical tests, you may upload a single table file with tests, Ns, etc., with reference to sections in the manuscript.)

**Group allocation**

- Indicate how samples were allocated into experimental groups (in the case of clinical studies, please specify allocation to treatment method); if randomization was used, please also state if restricted randomization was applied
- Indicate if masking was used during group allocation, data collection and/or data analysis

Please outline where this information can be found within the submission (e.g., sections or figure legends), or explain why this information doesn’t apply to your submission:

We did not perform group allocation, thus this information does not apply to our submission.

**Additional data files (“source data”)**

- We encourage you to upload relevant additional data files, such as numerical data that are represented as a graph in a figure, or as a summary table
- Where provided, these should be in the most useful format, and they can be uploaded as “Source data” files linked to a main figure or table
- Include model definition files including the full list of parameters used
- Include code used for data analysis (e.g., R, MatLab)
- Avoid stating that data files are “available upon request”

Please indicate the figures or tables for which source data files have been provided:

We have uploaded a summary data table for our screen as table S1.
